# General strength and conditioning versus motor control with manual therapy for improving depressive symptoms in chronic low back pain: A randomised feasibility trial

**DOI:** 10.1371/journal.pone.0220442

**Published:** 2019-08-01

**Authors:** Megan Teychenne, Karen E. Lamb, Luana Main, Clint Miller, Andrew Hahne, Jon Ford, Simon Rosenbaum, Daniel Belavy

**Affiliations:** 1 Deakin University, Geelong, Australia, Institute for Physical Activity and Nutrition (IPAN), School of Exercise and Nutrition Sciences; 2 Murdoch Children’s Research Institute, Royal Children’s Hospital, Parkville, Australia; 3 Department of Paediatrics, The University of Melbourne, Parkville, Australia; 4 Deakin University, Geelong, Australia, School of Exercise and Nutrition Sciences; 5 La Trobe University, Melbourne, Australia; 6 School of Psychiatry, University of New South Wales, Sydney, Australia; 7 Black Dog Institute, Sydney, Australia; Brown University, UNITED STATES

## Abstract

**Objectives:**

Exercise can be used as a treatment for depressive symptoms in the general population. However, little is known as to whether exercise has mental health benefits for adults experiencing chronic low back pain (CLBP). The aim of this study was to examine the feasibility of two intervention protocols commonly used in clinical practice for treating chronic low back pain, but with differing exercise dose, on depressive symptoms.

**Methods:**

Forty men and women (mean age = 35) experiencing chronic persistent (>3 months), non-specific low back pain were recruited into a randomised clinical trial during 2015–2016. Participants were randomised to receive motor control (low-dose exercise) and manual therapy (n = 20), or general strength and conditioning training (moderate-dose exercise) (n = 20). Depressive symptoms were assessed fortnightly throughout a 6-month follow-up period using the Centre for Epidemiologic Studies Depression Scale (CES-D 10). Linear mixed models were used to examine within-group and between-group changes in depressive symptoms.

**Results:**

Mean CES-D 10 score at baseline was 9.17 (SD = 4.32). There was evidence of a small decrease in average depressive symptoms over time (β -0.19 per fortnight, 95% CI = -0.34, -0.02). However, there was no evidence that change over time was dependent on treatment group.

**Conclusions:**

Reduction in depressive symptoms amongst adults with CLBP occurred with both treatment methods (motor control [low-dose exercise] and manual therapy; or general strength and conditioning [moderate-dose exercise]). Further interventions including a true control group are needed to draw conclusions as to the effectiveness of each of these treatment methods on depressive symptoms amongst adults with CLBP.

**Trial registration:**

Australian New Zealand Clinical Trials Registry, ACTRN12615001270505. Registered on 20 November 2015.

## Introduction

Globally, approximately 322 million people experience clinically diagnosed depression [1]. This mental illness impacts on various life domains including work productivity [2], personal relationships, and overall quality of life [3], and increases risk of suffering co-morbidities including cardiovascular disease [4], anxiety [5], and pain symptoms [6]. Adults experiencing chronic non-specific low back pain (CLBP) are three to four times more likely to suffer from clinical depression compared to the general population [7]; whilst clinical depression in adults experiencing chronic pain is linked to greater pain intensity, longer duration of pain and more pain complaints [6]. Further, among adults who have previously experienced low back pain, those with depression or heightened depressive symptoms have a greater chance of developing new episodes of lower back pain compared to non-depressed individuals [8]. Therefore, it is important to identify and test strategies to reduce the risk of clinical depression and/or depressive symptoms amongst those with CLBP.

Treatment modalities for depression (both clinically diagnosed and heightened depressive symptoms) in adults experiencing CLBP include pharmacological (e.g. anti-depressant medication) and psychological therapy [6]. However, non-compliance, cost and potential side-effects can limit uptake and adherence to these methods. Another less explored depressive symptom treatment for adults experiencing CLBP may be exercise. In the general population (i.e. those not experiencing chronic pain), exercise has been established as having a large effect on reducing depressive symptoms [9]. A review of 25 randomised controlled trials (RCT’s) showed that interventions that included either aerobic exercise or mixed (aerobic and resistance training) modes of moderate to vigorous intensity, and were supervised by exercise professionals had the largest effects on reducing depressive symptoms [9]. An increasing body of evidence has indicated that resistance training can also reduce depressive symptoms amongst adults experiencing minor or major depression [10], although relatively few studies exist.

There is limited evidence of the effectiveness of exercise interventions for improving mental health (e.g. depressive symptoms) among populations experiencing chronic pain (such as those with CLBP), and of the few studies that do exist, findings have been mixed. [11]. For example, studies have shown that aerobic exercise programs resulted in decreased long-term depressive symptoms in adults with fibromyalgia (i.e. a disease characterised by extensive pain) and those experiencing low back pain respectively [12, 13]. In contrast, Focht et al found that walking and strengthening exercises did not improve acute psychological well-being amongst obese older adults with knee osteoarthritis [14]. Given the possible mental health benefits exercise may induce amongst individuals with CLBP, further studies are needed to determine the effectiveness of exercise for reducing depressive symptoms in this population group.

There is some evidence to show that manual therapy (i.e. a specialised field of physical therapy used for management/treatment of neuro-muscular issues [15]) may result in a moderate reduction of depressive symptoms amongst patients with tension-type headache [16]. Among adults with CLBP, manual therapy has been shown to improve function and reduce pain [17], which could theoretically lead to improvements in depressive symptoms. Since individuals with CLBP are likely to receive manual therapy (typically delivered in conjunction with motor control exercise) for the treatment of back pain, utilising this method as an approach to reduce depressive symptoms in this high-risk target group may hold promise. Yet the evidence-base regarding the efficacy of manual therapy/motor control exercise on treating depression/depressive symptoms is currently limited [16]. Therefore, the aim of this study was to examine the feasibility of two different treatment protocols typically used for treatment of back pain (motor control [low-dose exercise] and manual therapy; or general strength and conditioning [moderate-dose exercise]) for improving depressive symptoms in adults with CLBP.

## Methods

Analyses in this study were based on data collected formerly from a trial exploring a range of other primary outcomes [18].

### Study design

This randomised feasibility trial with a 6-month follow-up comprised two exercise treatment (intervention) groups (motor control and manual therapy, MCMT; or general strength and conditioning, GSC). Repeated assessments were performed at baseline, 3- and 6-months, as well as fortnightly assessments on selected variables. The study was approved by the Deakin University Human Research Ethics Committee and registered with the Australian New Zealand Clinical Trials Registry (ACTRN12615001270505; Date registered 20/11/2015). The full study protocol has been published previously [18] and is summarised briefly below. There were no changes to methods or trial outcomes after the trial commenced and no unintended effects as a result of the trial.

### Participants

In November 2015 to December 2016 participants were recruited into this study, based on the following inclusion criteria: 1) men and women (aged 25–45 years), 2)living in the inner and Eastern suburbs of Melbourne, Australia, 3) experiencing non-specific chronic (>3 months) low back pain (located between the T12 vertebra and gluteal fold). The age range 25–45 years was selected due to one of the trial’s primary outcomes measuring disc degeneration. Various recruitment methods were utilised to identify participants, including: print and web-based advertising (including in local businesses and medical centres located in inner and Eastern suburbs of Melbourne), emails sent to University staff and students, social media posts (including Twitter and Facebook), and word of mouth. Potential participants registered their interest via the study website and were then screened for eligibility by a researcher via telephone according the specified inclusion/ exclusion criteria (see Table 1).

**Table 1:** Exclusion criteria for the study.

Exclusion criteria
• History of or planning (within 6 months) invasive spinal surgery
• Had a previous traumatic spinal injury
• Currently experiencing symptoms of cauda equine syndrome or nerve root compression
• Diagnosed with structural scoliosis
• Currently receiving treatment for back pain
• Undertaking more than one day per week of gym-based exercise or organised sport
• Currently meeting the physical activity guidelines of 150 minutes per week of moderate-vigorous physical activity
• Unable to communicate in English
• Has a compensable claim for their back pain
• Currently or possibly pregnant or planning a pregnancy within 6 months
• Less than 9 months postpartum or currently breastfeeding
• Currently smoke
• Has known anaemia
• Weigh more than 120kg
• History of seizures or epilepsy, stroke, head injury or brain-related disorders
• Currently taking medication for any mental illness
• Unable to commit to the full program and assessment days (including those who were unsuitable for an MRI such as having metal or electronic implants or had nuclear medicine in the previous 3 months)

This study is based on an exploratory analysis of a secondary outcome. Sample size calculations for the trial were conducted for the primary outcome (lumbar IVD T2-time). These sample size calculations indicated that 40 participants (20 in each arm) were required to detect a difference between groups of 0.5%, or an effect size of 0.041 (alpha = 0.05, power = 0.80), assuming a 10% loss to follow-up. This was based on a continuous outcome, measured at three time points. Using the formula of Ahn et al. [19], a sample size of 40 (or 36 allowing for the 10% loss to follow-up) was found to provide 80% power to detect a minimum difference in group slopes (i.e., coefficient of time for each of the trial arms) of between 0.29 and 0.62, assuming 13 measurement occasions for each participant (as was planned in the trial for the CESD-10 outcome) and standard deviations of 5.5 for the outcome CESD-10 (based on data from Teychenne et al. [20]) and 3.74 for time (based on the proposed 13 time-points of data collection). Due to uncertainty about the sizes of the within-subject correlation and ratio of the random slope variance to the sum of the other variance terms required, these parameters were allowed to vary in sample size calculations. Within-subject correlations of 0.2, 0.4 and 0.6 and ratios of 0.001, 0.005 and 0.01 were considered, based on estimates from other studies[20], resulting in the range in minimum detectable difference presented above.

**Fig 1:**
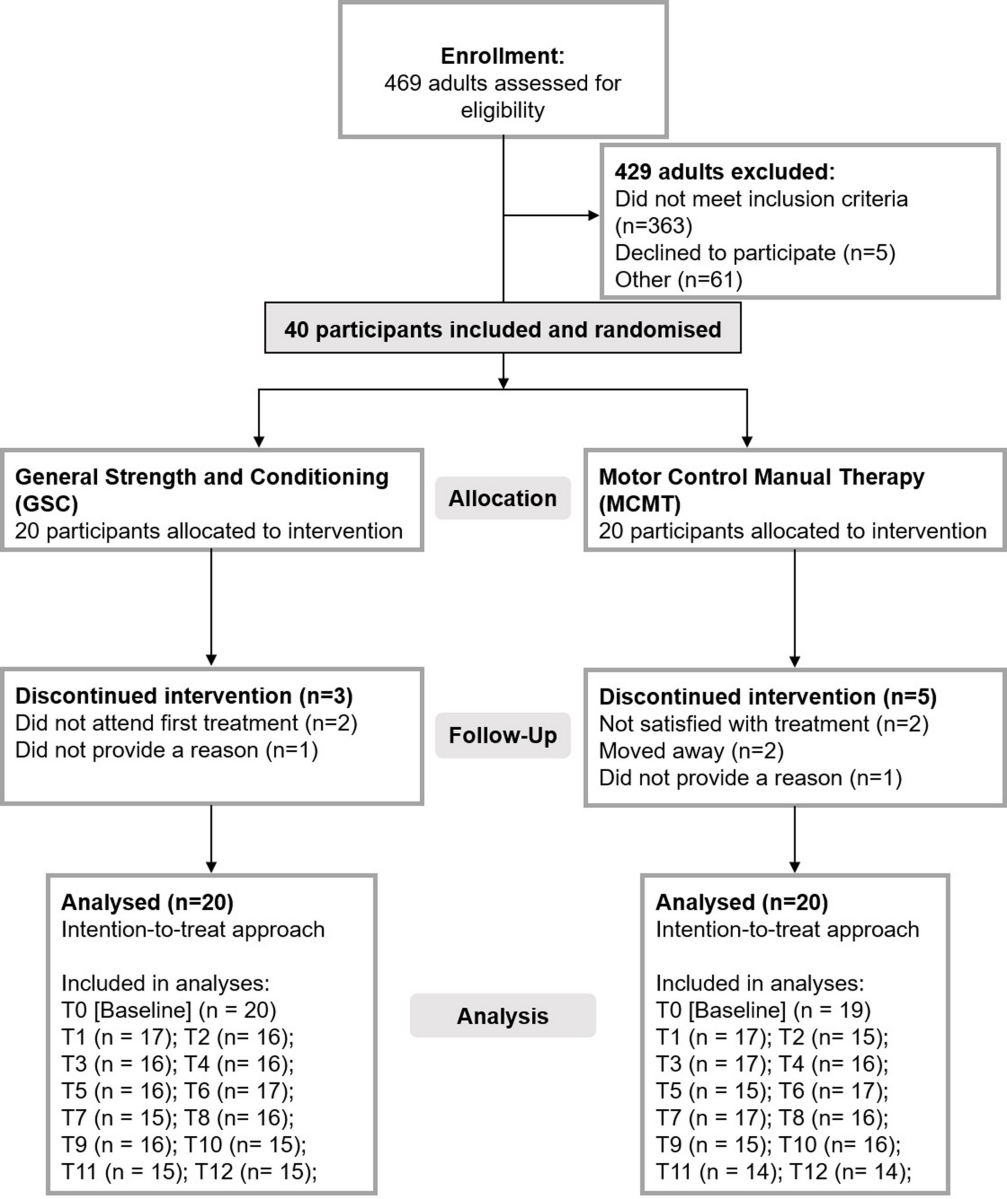
Participant recruitment flowchart.

Of the 469 interested participants, 40 (21 men and 19 women) met full entry criteria and agreed to participate (Fig 1). Written consent was provided by all participants.

### Randomisation

Randomisation was performed by an offsite researcher external to the University and the treatment sites to minimise contamination. Block randomisation (with block lengths of 4 or 6 in random order, and stratifying by sex) was conducted, to maximise the chance that equal numbers of participants would be allocated to each treatment group (i.e. MCMT or GSC) in equal gender proportions. Concealed allocation was then implemented by the offsite researcher. Blinding of participants or therapists was not possible given the nature of the treatment.

### Interventions

#### Motor control (low-dose exercise) and manual therapy (MCMT)

Participants in the MCMT group (n = 20) undertook 12 x 30-minute physiotherapy sessions (one-to-one with a physiotherapist) following established protocols for treatment of chronic low back pain [21]. A total of 10 x 30 minute sessions were delivered in the first three months (approximately weekly sessions), with two sessions in the final three months spaced every 4–6 weeks. Frequency of treatment sessions was determined as clinically relevant and tailored to the participant, which represents an ecologically valid clinical treatment approach. Physiotherapy sessions included pain education plus: 1) Motor control exercises targeted local/deep trunk muscles including transversus abdominis, lumbar multifidus, and the pelvic floor muscles. These were commenced in unloaded positions, and then progressed to upright and functional activities, with progression occurring on a pain-contingent basis [21–23] (Table A in S1 File). Exercises were taught and practiced during the treatment sessions and also in a structured home based program; 2) Manual therapy was administered in accordance with commonly used principles [24], with techniques including joint mobilisation and soft techniques of the lumbo-pelvic area.

#### General strength and conditioning (moderate-dose exercise) (GSC)

Participants in the GSC group (n = 20) received pain education and undertook gym-based (supervised by an exercise physiologist) and home-based (unsupervised) exercise sessions. Each gym-based session was one-hour in duration, with participants required to attend two sessions per week (during the first three months), and then for the final three months participants could self-select to attend either one or two supervised training session per week. Exercises included aerobic conditioning (20 minutes at an intensity of 65–85% HR_max_), proprioceptive exercises (i.e. general balance [e.g. single leg with single arm lateral raise], weight transfer [e.g. single leg dumbbell transfer left to right] and external perturbation [e.g. medicine ball chest pass]) and progressive resistance training. Exercises are described in Tables B and C in S1 File. The 20 minutes of aerobic conditioning was inclusive of a warm-up, with a progressive increase in intensity over the first five minutes. The resistance training program included a selection of seven exercises focussed on push, pull, lift, trunk extension and trunk flexion. Each set was completed to volitional fatigue and progressed by increasing load within each phase after successful completion of all sets at the maximum repetition range and progressed in a time-contingent manner and not modified in response to daily changes in reported pain. Each phase was structured using an undulating periodization approach. Phases varied by exercise selection, relative load, sets, repetition range and time under tension. Participants were additionally required to undertake mental rehearsal exercises at home which focussed on activities that individuals perceived as pain-provoking for five minutes every day (for the first six weeks). Exercise tasks which replicated these movements were then introduced into the supervised gym-based (resistance training) program. Home-based exercise sessions (unsupervised) involved 20–40 minutes of aerobic exercise (e.g. walking or jogging at 65–85% HR_max_), followed by stretching (based on individuals need), up to three times per week.

### Measures

All self-report data in this study were collected via online questionnaires. Age and sex demographic data were assessed at baseline using a self-report survey. Baseline back pain was assessed using a visual analogue scale (VAS) whereby participants rated their back pain on average over the past week on a scale of 0–100.

#### Depressive symptoms

Depressive symptoms were assessed every two weeks (from baseline through to 24 weeks; T0—T12) using the 10-item Centre for Epidemiologic Studies Depression Scale (CES-D 10) [25]. The CES-D 10 is a validated self-report measure of depressive symptomology [26], whereby participants indicated how often they experienced various symptoms of depression in the past week using a 4-point severity scale (ranging from “rarely or none of the time” to “most or all of the time”). Responses were coded and summed as per protocol (possible range 0–30), with scores of 10 or greater indicating being ‘at risk’ of depression. Since there was a delay of up to 3 weeks between baseline testing (B1) and randomisation, baseline data collection was conducted twice for some participants (B1 and B2, with B2 being conducted immediately prior to randomisation) and the value closest to the randomisation date was used as the baseline value to calculate change from baseline. In the situation where B2 was missing, B1 was used as the baseline data.

### Statistical analyses

Analyses were conducted following an intention-to-treat approach using Stata statistical software (version 15.0). A linear mixed model was used to examine changes in depressive symptoms accounting for the clustering of observations within individuals. The model included time (fortnight), treatment group (MCMT or GSC), and a group-by-time interaction. The interaction between time and treatment group enabled the assessment of whether change in depressive symptoms from baseline varied depending on treatment group. The mixed model included a random intercept to allow for random variation in baseline depressive symptoms between participants and a random slope for time to allow for random variation in the change in depressive symptoms over time between participants. Covariation between the random intercept and slope was assessed to determine if the change over time was dependent on the baseline level of depressive symptoms. Model assumptions were assessed by examining plots of the residuals. Accepted significance level was set as p<0.05.

#### Missing data

Of the 40 participants included in this study, 16 (40%) had complete outcome (CES-D 10) data across the 13 data collection points. Data were analysed on a missing at random assumption. Mixed models are found to perform well in comparison to other methods of handling missing data [27]. Therefore, all available data was utilised (even if a participant did not provide depressive symptoms information for all time points), since mixed models handle imbalance in the number of observations.

## Results

The final sample consisted of 40 participants (MCMT = 20; GSC = 20) with data for inclusion in analyses, although 8 participants (MCMT = 5; GSC = 3) withdrew from the study. Reasons for withdrawal are presented in Fig 1. Average age and level of back pain at baseline was comparable between the two treatment arms and the number of females was similar (Table 2).

**Table 2:** Demographic characteristics of the MCMT and GSC intervention groups at baseline.

	Motor control and manual therapy	General strength and conditioning
N	20	20
Age (years; mean ± sd)	34.6 ± 3.66	34.8 ± 4.91
Women, n (%)	10 (50%)	9 (45%)
Back pain score (mean, SD)	49.61 ± 17.41	42.03 ± 17.35
CESD-10 score (mean, SD)	8.16 ± 3.99	10.15 ± 4.50
At risk of depression (CESD-10 ≥ 10) n (%)	7 (35%)	10 (50%)

**Fig 2:**
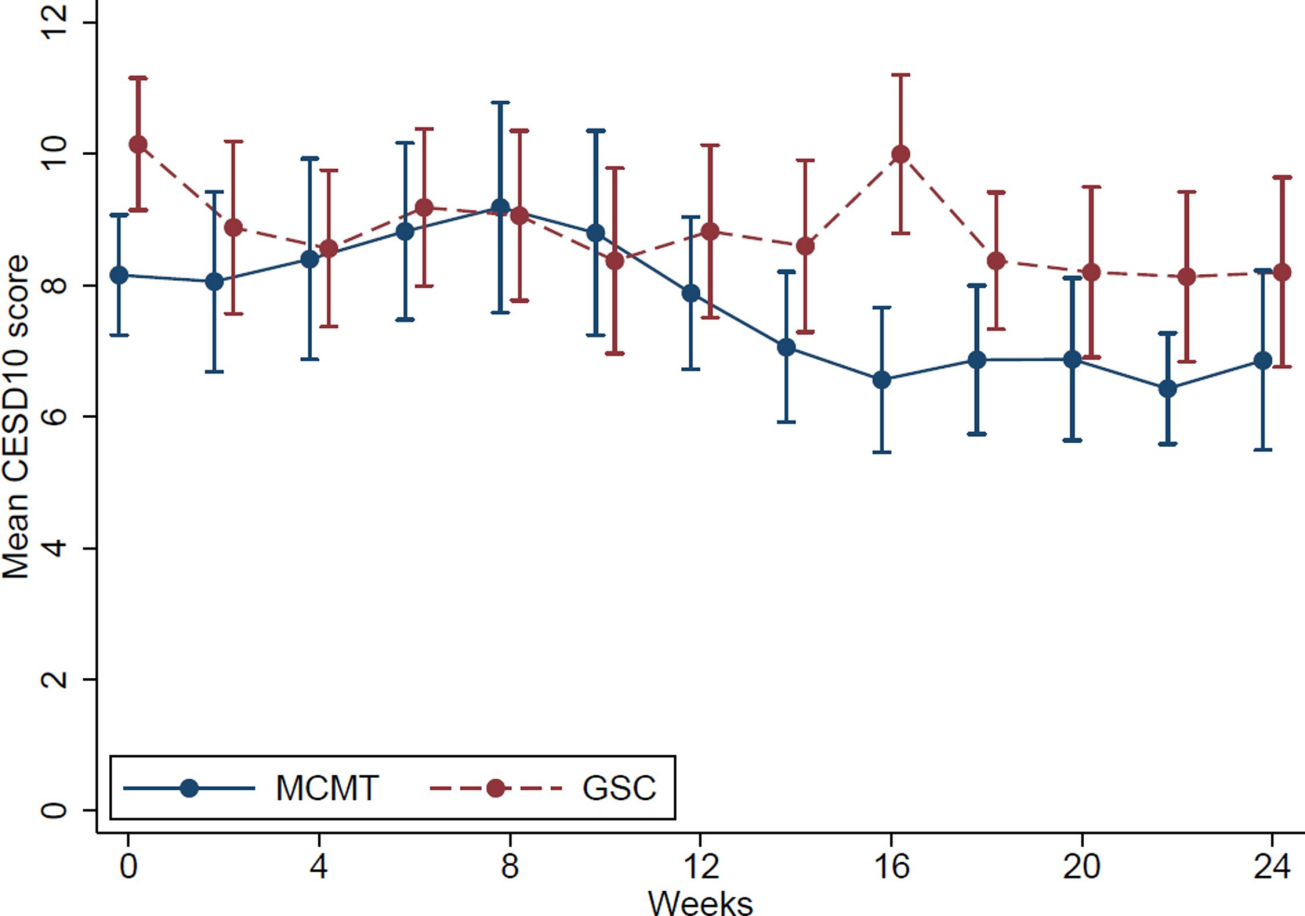
Observed mean CES-D 10 values at each time point for the two groups. Error bars show +/- 1 standard error. Legend: MCMT, Motor control and manual therapy; GSC, General strength and conditioning.

Fig 2 shows observed mean CES-D 10 values at each time point for the two groups.

Table 3 presents inferential results for the change over time in depressive symptoms. There was no evidence of a difference between groups for change in depressive symptoms over time. The MCMT group (reference) demonstrated a slope of -0.19 (equating to -2.28 by the end of the trial (24 weeks)), while the GSC group demonstrated a slope of -0.10, 95% = -0.25. 0.04 (equating to -1.24 by the end of the trial) for depressive symptoms (per fortnight) (between-group difference = 0.09, 95% CI = -0.12, 0.30). Further, there was no correlation for intercept and slope (i.e. change over time did not vary by baseline depressive symptoms).

**Table 3:** Linear mixed model examining change over time in depressive symptoms.

Covariate	β (95% CI)
Intercept	8.95 (7.11, 10.79)
Time (per fortnight)	-0.19 (-0.34, -0.02)
Group*	0.53 (-2.06, 3.13)
Group* x time	0.09 (-0.12, 0.30)

* Reference group = Motor control manual therapy (MCMT)

## Discussion

Participants undertaking motor control (low-dose) exercise and manual therapy or general strength and conditioning (moderate-dose exercise) showed small reductions in depressive symptoms over the 6-month study period. However, these changes did not differ between intervention groups. Although previous research is limited, the finding in the current study that depressive symptoms decreased over time for those undertaking an exercise program is consistent with a number of previous controlled trials amongst adults experiencing chronic pain (e.g. fibromyalgia and CLBP) [12, 13]. Given that a large body of evidence in the general population suggests that exercise (including a mix of aerobic and strength training) can be used as a treatment for clinical depression and/or depressive symptoms [9], collectively our findings may indicate that exercise can also be used safely as a treatment (or adjunct or adjuvant treatment) for depressive symptoms in adults with CLBP. However, since the magnitude of change in depressive symptoms was small (i.e. change in depressive symptoms for the exercise group equated to a -1.2 reduction on the CES-D 10 scale after 24 weeks), and that the current study did lack a true control group, these findings need to be confirmed in future RCT’s.

To our knowledge, this is one of the first studies to examine the addition of manual therapy to motor control exercises for treating depressive symptoms. Similarly, previous studies examining the effect of manual therapy alone on depressive symptoms are currently limited. From the small body of existing research, there is some indication that manual therapy may lead to a reduction of depressive symptoms amongst adults experiencing tension-type headache [16], which is consistent with the findings of the current study. However, it should be acknowledged that the magnitude of change in depressive symptoms for the MCMT group was small (i.e. change in depressive symptoms equated to a -2.28 reduction on the CES-D 10 scale after 24 weeks). Given that the manual therapy intervention in the current study also included motor control exercises, these findings highlight a potential avenue to explore manual therapy as an adjunct therapy option for improving depressive symptoms amongst patients with CLBP, particularly since they are a target group already at heightened risk for depression [7]. Given that manual therapy and motor control exercise is often delivered concurrently by allied health professionals, this ‘combined’ treatment protocol reflects real-world application. Although we are unable to attribute changes in depressive symptoms to either motor control exercise OR manual therapy, changes may be attributed to this combined therapy approach, typically used together as a treatment (for back pain) for people with CLBP.

Given that participants in both intervention groups (i.e. motor control [low-dose exercise] and manual therapy, and general strength and conditioning [moderate-dose exercise]) exhibited a reduction in depressive symptoms over the 6-month intervention period, it is important to recognise the potential mechanisms that may explain these mental health changes. Firstly, both interventions included exercise as a key component. Although the dose of exercise prescribed to participants varied (with the MCMT group receiving much lower intensity and volume of exercise), studies have shown that even exercise of a low dose (e.g. light to moderate intensity, short duration) may be effective reducing current depressive symptomatology [28] and in reducing risk of developing future depressive episodes in the general population [29]. Exercise has been hypothesised to improve mental health through various potential biological mechanisms including altering neurotransmitters (e.g. serotonin receptors (5-HT)), hormones (e.g. norepinephrine (NE)), and proteins in the brain, (e.g. brain-derived neurotrophic factor (BDNF)) [30, 31], as well as reducing pro-inflammatory cytokine levels [32], which influence the 5-HT system [33]. Such pathways may explain the reduction in depressive symptoms observed in the current study. An alternative explanation is the ‘social interaction’ hypothesis, which suggests that mental health may be enhanced through social interaction, which both intervention groups were exposed to (i.e. with the exercise physiologist or physiotherapist). Alternatively, both treatments were aimed to reduce pain and given that chronic pain is a key determinant of depressive symptoms [6], it could be that the reduction in depressive symptoms was a result of associated pain relief. However, mediating analyses using larger samples are needed to test these hypotheses.

### Strengths and limitations

This was an exploratory study. Limitations of this study included a small sample size, which may limit generalisability of results. It was designed to provide 80% power to detect minimal differences in depressive symptoms between groups. Since there was no true control group, causality could not be determined. It is possible that other factors such as seasonal variation or the utilisation of therapies such as psychotherapy or counselling could potentially partly explain changes in depressive symptoms over time. Depressive symptoms were assessed using a self-report measure and therefore may be subject to socially desirable responses. Further, baseline depressive symptoms were relatively low for both groups with nearly 60% of participants being classified as “not at risk” of depression (i.e. scoring less than 10 on the CES-D 10). Therefore, it is unknown whether the pattern of change in depressive symptoms experienced by a clinically depressed group completing either intervention would be the same as what was observed in this study. The dose and/or time of intervention conditions was not the same for all participants. For example, participants in the GSC group could self-select either one or two training sessions per week during the final three months. Similarly, participants in the MCMT group were prescribed two sessions in the final three months, with frequency varying based on individual’s clinical need and availability. However, both intervention conditions were designed to reflect clinical practice (e.g. clients would typically undergo individual consultations with clinicians and prescribed a dose of treatment tailored to them) so that findings can be feasibly applied in real world settings. A strength of this study was that the screening process identified and excluded those on anti-depressant medication, and those who were pregnant or postpartum, which are all factors that could influence depressive symptoms. Furthermore, both interventions included both clinic (supervised) and home-based (unsupervised) settings, which may increase initial engagement and long-term adherence to such programs in the real-world setting [34]. Finally, very few studies have examined changes in depressive symptoms following different exercise protocols in people with CLBP. Given this group are at heightened risk of experiencing poor mental health, these findings provide new insights into potential treatment options for this high-risk target group.

In conclusion, adults with CLBP experienced a reduction in depressive symptoms when undertaking either motor control and manual therapy; or general strength and conditioning. Further RCT’s (including those using a true control group) are needed to draw conclusions as to the effectiveness of each of these treatment methods in this population group.

## Supporting information

S1 File Supplementary tables. (DOCX)

S2 File Final approved plan language statement. (PDF)

S3 File CONSORT checklist. (DOC)
