# Supplementary material for: General strength and conditioning versus motor control with manual therapy for improving depressive symptoms in chronic low back pain: A randomised feasibility trial
Source: PLoS One. 2019 Aug 1;14(8):e0220442. doi: 10.1371/journal.pone.0220442 (PMC6675067; doi:10.1371/journal.pone.0220442)
Supplement: S1 File — (DOCX) [file pone.0220442.s001.docx]

Supplemental Table A. Motor control exercise plus manual therapy (MCMT): Exercise progressions

|  | Progression | | | | |  |
| --- | --- | --- | --- | --- | --- | --- |
|  | **1** | **2** | **3** | **4** | **5** | |
| **Exercise** | Side-lying transverse abdominis activation | Four-point transverse abdominis activation | Standing transverse abdominis activation | Walking transverse abdominis contractions | Transverse abdominis activation during functional loading task (e.g. stair climbing) | |
| **Dose** | 5 x 5s hold four times a day | 5 x 5s hold four times a day | 5 x 5s hold four times a day | Start at 2-min of 10s activation on/off | - | |
| **Progression criteria** | Progress position if good control and endurance – pain free during exercise | Progress position if good control and endurance – pain free during exercise | Progress if good control and endurance – pain free during exercise | Progress to 2-min hold before functional loading – pain free during task | - | |

Supplemental Table B. General strength and conditioning (GSC): Proprioceptive exercises

| Exercise type | Specific exercises | | | | | | | |
| --- | --- | --- | --- | --- | --- | --- | --- | --- |
|  | **1** | **2** | **3** | **4** | **5** | **6** | **7** | **8** |
| **Perturbation** | Ball Chest Pass | Medicine ball chest pass | Single arm throws and catch | Single leg medicine ball chest pass | Single leg single arm throws and catch | Single leg single arm throws and catch on dura-disc | Single arm and leg throws on foam | - |
| **Weight transfer** | Standing tandem dumbbell transfer left-right | Single leg dumbbell transfer left-right | Single leg single arm lateral raise | Single leg single arm medicine ball body contact target (hip, shoulder, head, waist, knee) | Single leg medicine ball around the world | Single leg single arm medicine ball body contact target (hip, shoulder, head, waist, knee) on foam | Alphabet with foot | Alphabet with foot on foam |
| **General balance** | Tandem stance; eyes open | Tandem stance; eyes closed | Single leg stance; eyes open | Single leg stance; eyes closed | Single leg stance on foam; eyes open | Single leg stance on foam; eyes closed | Single leg on dura-disc; eyes closed | - |

Supplemental Table C. General strength and conditioning (GSC): Resistance training exercises

| Exercise type | Specific exercises | | | | |
| --- | --- | --- | --- | --- | --- |
|  | **1** | **2** | **3** | **4** | **5** |
| **1 (Lift)** | Sit to stand | Touch downs | Body weight squats | Dumbbell squats | Back squats |
| **2 (Extension 1)** | Prone floor opposite arm and leg | Prone opposite arm and leg on swiss-ball | Prone trunk extension on swiss-ball | - | - |
| **3 (Extension 2)** | Supine double leg bridge | Supine single leg bridge | Supine double leg bridge on swiss-ball | Supine swiss-ball hamstring curls | Supine double leg bridge with weight plate |
| **4 (Extension 3)** | Standing latissimus dorsi (lat) pull down | Standing straight arm pull down | Modified deadlifts | Latissimus dorsi (seated) pull down | One-leg deadlift |
| **5 (Flexion)** | Partial curl up in supine fingers to knee | Partial curl up in supine wrist to knee | Bosu-ball crunches | - | - |
| **6 (Push)** | Split stance double arm cable chest press | Split stance single arm cable chest press | Single leg double arm chest press | Single arm opposite leg cable chest press | Bench press |
| **7 (Pull)** | Split stance double arm cable row | Split stance opposite arm cable row | Single leg double arm cable row | Single leg opposite arm cable row | Seated row |
