# Supplementary material for: General strength and conditioning versus motor control with manual therapy for improving depressive symptoms in chronic low back pain: A randomised feasibility trial
Source: PLoS One. 2019 Aug 1;14(8):e0220442. doi: 10.1371/journal.pone.0220442 (PMC6675067; doi:10.1371/journal.pone.0220442)
Supplement: S2 File — (PDF) [file pone.0220442.s002.pdf]

PLAIN LANGUAGE STATEMENT AND CONSENT FORM

TO: Participants

Plain Language Statement

Date: 21/07/2015

Full Project Title: Optimising conservative management of chronic low back pain

Principal Researcher: Associate Professor Daniel Belavy

Associate Researchers: Dr. Jon Ford **(Advance Healthcare)**, Dr. Clint Miller **(Deakin)**, Dr. Andrew Hahne **(Advance Healthcare)**, Dr. Luana Main **(Deakin)**, Dr. Timo Rantalainen **(Deakin)**, Dr. Wei-Peng Teo **(Deakin)**, Dr. Megan Teychenne **(Deakin)**, Professor Guy Trudel **(University of Ottawa, Canada)**, Professor Guoyan Zheng **(University of Bern, Switzerland)**, Professor Gary Thickbroom **(Burke-Cornell Medical Research Institute New York, USA)**

This Plain Language Statement and Consent form is 13 pages long. Please make sure you have all the pages.

Your Consent

You are invited to take part in this research project.

This Plain Language Statement contains detailed information about the research project. Its purpose is to explain to you as openly and clearly as possible all the procedures involved in this project so that you can make a fully informed decision whether you are going to participate.

Please read this Plain Language Statement carefully. Feel free to ask questions about any information in the document. You may also wish to discuss the project with a relative or friend or your local health worker. Please feel free to do this.

If you decide you want to take part in this project, we will ask you to obtain clearance from your GP.

Once you understand what the project is about and if you agree to take part in it, you will be asked to sign the Consent Form. By signing the Consent Form, you indicate that you understand the information and that you give your consent to participate in the research project.

Purpose

The overall aim of this study is about understanding how different kinds of treatment for chronic pain in the lower back affect the body.

If you are eligible for this study, you will be assigned, at random, to one of two treatment groups. One group will receive physiotherapy treatment at Advance Healthcare in Boronia over the course of 6 months. The second group will receive spine and general physical conditioning exercise over the course of 6 months at Deakin University.

To understand what changes occur in your body due to the different treatment approaches, a battery of tests will be performed at the start of the study, 3 months and 6 months. Magnetic resonance imaging, tests of muscle strength, performance and endurance, a scan of the bone in your spine, amount of fat, muscle and bone in different regions of your body, a series of questionnaires, and an assessment of how muscle activation occurs in the brain will be performed. Also, you will be asked to complete a short questionnaire every two weeks.

Methods and Demands: what you will be asked to do

If you participate in the study, you will undergo a **treatment program** at one of the following two facilities. Treatment (and testing) will typically occur during normal business hours. If you wish to participate in the study, **it is important that you can attend treatment during normal business hours**:

- Deakin University Burwood Campus, 221 Burwood Highway, Burwood VIC 3125. This treatment program will involve:
  - a progressive exercise program as part of 1-on-1 sessions, group sessions and a home exercise program aiming to improve their muscle strength, overall body endurance, posture, nutrition of the intervertebral disc and balance control
  - 1 hour (group) exercise sessions
  - 2 sessions per week in first 12 weeks
  - 1-2 session per week for last 12 weeks
  - A home exercise program with an exercise diary
  - Come to the Clinical Exercise Learning Centre (CELC) on Level 1 of Building J: https://goo.gl/maps/vyhqb
- Advance Healthcare Boronia, 157 Scoresby Road Boronia VIC 3155. This treatment will involve:
  - manual therapy (gentle massaging and pressing on your back to reduce pain and stiffness) and motor control training (exercises aimed at strengthening the low stomach muscles and deep back muscles, sometimes called "core strengthening")
  - 12 thirty minute treatment sessions with a physiotherapist over 6 months
  - A home exercise program with an exercise diary
  - The location of this treatment centre can be found here: https://goo.gl/maps/XXAkp

You cannot choose which group you will participate in.

Even if your pain resolves, it is expected that you continue with the treatment program.

If you are eligible for the study, you will be randomised (assigned by chance) to one of the two groups. If you are not **certain that you can attend treatment** as listed above for the **full 6 month period** of the study, then you **should not participate in this study**.

As part of the study, you will also undergo a **testing program** to assess how you are progressing and how your body changes over the course of the 6 months treatment. These testing sessions will be spread over **2 days** and will be done **three times over the 6 month period** of the study.

You will need to attend Deakin University Burwood Campus (Building J, Level 5 Research Facility) for **3 hours and** also attend Swinburne University for an **approximately 1.5 hour MRI** for a total of **three times in the 6 months period** of the study. Furthermore, you will need to complete online **questionnaires every two weeks**. If you are not certain you **can commit to this time investment** or are unsure your pain in your lower back might prevent you from completing this testing regime, you **should not participate in the study**.

The **testing program will always occur during normal business hours**. Hence, if you are not sure you can attend these sessions, you should not participate in the study.

The following tests will be performed at Swinburne University:

- A **magnetic resonance imaging** (MRI) scan will be used to measure the discs, muscles, bones, fat content, water content and your ability to contract the muscles of your back and stomach.

The MR scanning centre at Swinburne University is located in the ATC building on Burwood road (See map below. Arrow indicates the building location).

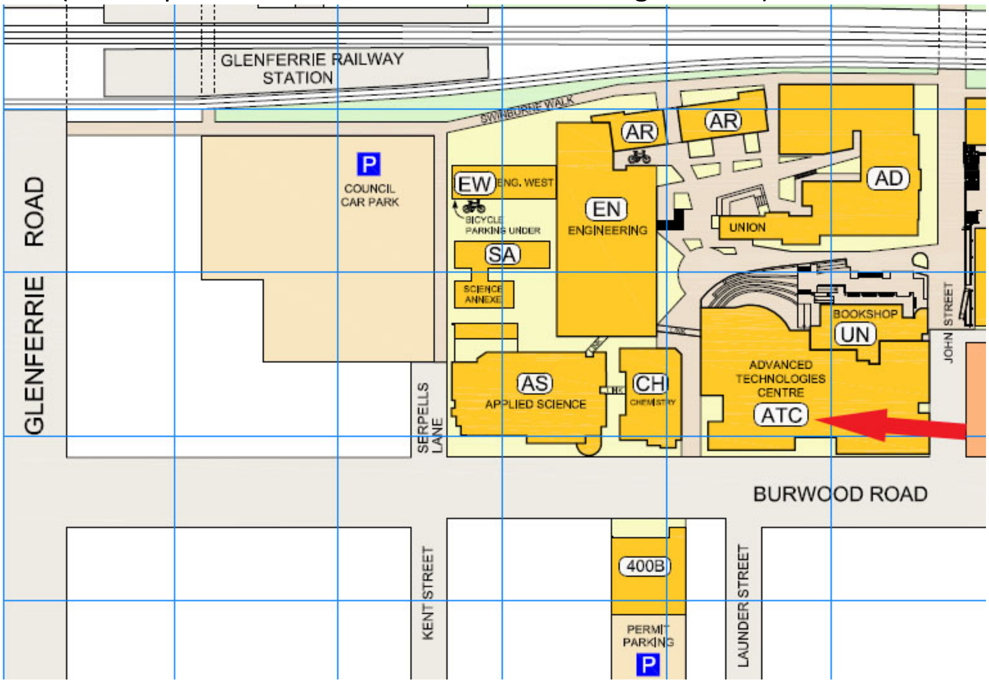

- Clothing: please wear or bring with you tracksuit pants or shorts and a loose shirt (ensure there is no metal in these clothes which you will wear in the MR). For women, please ensure bras are without metal underwires or clips.
- Glenferrie Railway station is nearby and there are some free and paid parking zones nearby. If you pay for parking, please keep the receipt so we can reimburse your costs.
- When you enter this building, take the lifts or the stairs down to the lower ground floor. You will be greeted by study staff. If you are not sure where to go, you can also dial 5514 on an internal phone (at main desk at entrance to building or on the lower ground floor) or 9214 5514 from your own phone to reach the MR control room.
- Prior to the scan, you will need to sit for 20 minutes. If this is your first appointment for the study, please allow approximately 1 hour before the scan appointment for completing study forms and questionnaires.
- In addition to travel time, please budget approximately 1.5 hours (2.5 hours if this is your first appointment) in total.
- MR scan appointments are possible between 9am and 3pm.

Please note: as part of standard protocols at Swinburne University, your MRI scans will be reviewed by a radiologist. If there are any findings that require medical review, you will be referred back to your GP for review prior to continuing with the study.

The following tests will be performed at Deakin University Burwood Campus:

- Your **physical function and performance** will be measured: your one repetition maximum (1RM) strength will be measured by lifting a weight that progressively increases in weight until you are only able to lift it for one repetition. This will be done for leg press and for back extension. A test for muscular endurance will be performed based on your 1RM result. You will be required to lift 70% of your 1RM as many times as possible.
- Your **endurance capacity** will be measured in a graded test on a treadmill. You will undertake a walking test on a treadmill until you reach fatigue. The initial walking speed will be individually determined as equivalent to 'very easy' in effort. This speed will remain constant throughout the test and then gradient ("hill") will be increased each minute until you reach fatigue (not exhaustion). During this test, you will breathe through a mouthpiece; and heart rate, blood pressure, and oxygen content in your blood (by using a non-invasive infrared probe on a finger) will be recorded. If clinically necessary heart rate and rhythm using an ECG (electrodes attached to the skin on your chest) may be recorded. Typically, the test lasts 4 to 12 minutes.
- Measurements of your **body size** (such as height, waist size and hip size) and weight will be taken.
- **Dual X-ray absorptiometry** (DEXA) will be used to measure the bone in your lower spine and also the amount of muscle, fat and bone in different parts of your body.
- You will be given a series of **questionnaires** to evaluate, for example, your pain, how much your back pain prevents you from doing certain things, your mood, physical activity and function.

- **Transcranial magnetic stimulation** (TMS) will be used to assess how effectively your brain activates your muscles. This procedure involves the use of a magnetic field to induce current in the brain, which in turn causes a brief muscle twitch. When receiving TMS, the researcher will place a magnetic coil above the region of your brain that produces muscle contractions. When the researcher discharges the coil, you will hear a 'click' and may feel a light tap on your scalp underneath the coil. This will cause an involuntary muscle contraction, or twitch, which is recorded by small electrodes placed on the skin. A signal depicting the muscle contraction will appear on the computer screen, allowing the researcher to determine any changes in your muscle activation levels.

To get to the Level 5 Clinical Research Facility in Building J:

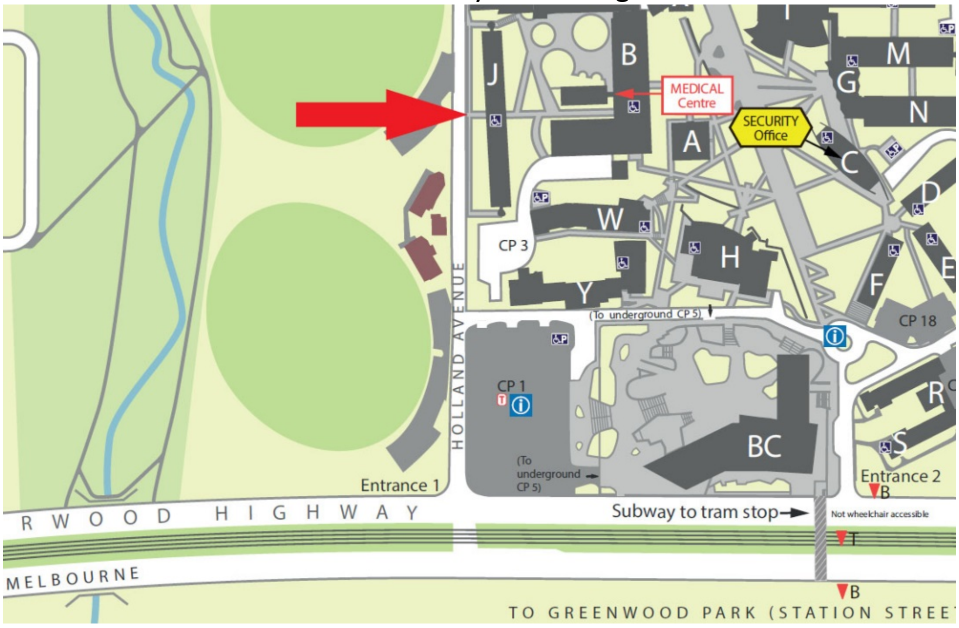

- When you come to Deakin University, walk to building J (arrow marked above).
- When you enter this building, the take the lift or the stairs up to level 5.
- Find the place in the foyer on level 5 where there is a sofa. You will be greeted here by study staff.
- If you park on-site, we can reimburse the cost of your parking (please keep the receipt).

Please note: if you are unable to complete all of the testing battery, you cannot participate in the treatment program.

Potential benefits to participants

We cannot guarantee or promise that you will receive any benefits from this project.

However, we expect that participants in both groups will benefit from their involvement in the treatment program. We also expect improvements in pain, function, muscle function, muscle strength.

Furthermore, at the end of the 6 month project, if you wish we can provide you with the results from your testing. This is access to information on your own body that you would otherwise not have.

It is possible that in the course of the study we might obtain information, such as from MRI, which might be of clinical significance. In this case we will inform you and advise you to talk to your GP.

Potential risks to participants

There are, however, some potential risks of the treatment program and/or testing program which you should consider before deciding to commit to this study. Some groups of people are at higher risk of negative side effects due to the study (e.g. pregnancy, certain types of metallic objects in some objects in some body regions) and will be excluded from the study.

- Treatment interventions: It is possible that some participants will experience some minor discomfort due to their performance of the exercises and/or manual therapy intervention. 'Treatment soreness' can occur as a normal part of treatment. General muscle soreness due to exercise is also possible. This is a normal response to progressive overload exercise and dissipates within a few days. There is small possibility that some participants in either treatment group will experience deterioration in their condition over time that is not necessarily related to the intervention.
- Magnetic resonance imaging (MRI): MRI scanning has been in use as a medical imaging tool for many years and with proper safety controls is commonly regarded by clinicians as a safe procedure. It does not employ ionising radiation (such as x-rays). It does however entail exposure to electromagnetic fields (EMF) which are much higher than levels recommended by international safety guidelines for general exposure (though still within limits of special guidelines for MRI scanning). Very occasionally, these EMFs may cause some tingling or heating sensations. These effects do not persist after scanning and have no known long term impact on health. Your MRI exposure will be carefully controlled to avoid such effects, and you will be constantly monitored for any signs of these effects and may direct us to stop the scan at any time if you experience uncomfortable sensations. The staff on duty will answer any queries you might have on the day, or if in doubt, call our department before your appointment. Some people cannot undertake MR scanning. Specific exclusion criteria are implemented for the study to ensure that people for whom MR scanning may be potentially unsafe. We have attached an information sheet about MR scanning from Swinburne University. You will be asked to complete a safety questionnaire by the radiographer on duty and they will determine whether you can safely enter MRI.
- Physical function and performance: you may experience some muscle soreness in the days after testing. This is a normal response of the body to strength and endurance testing.
- Endurance capacity: muscle soreness due to the exercise is possible. This is a normal response of the body to exercise and dissipates after a few days.
- Body size and mass measurements: no known risks
- DEXA: This research study involves exposure to a very small amount of radiation from DEXA scans of your body. As part of everyday living, everyone is exposed to naturally

occurring background radiation and receives a dose of about 2 millisieverts (mSv) each year. The effective dose you will receive from all the DEXA scans of your body will be approximately 0.05 mSv. At these dose levels, no harmful effects of radiation have been demonstrated, as any effect is too small to measure. The risk is believed to be very low. If you have been involved in any other research studies that involve radiation, please inform us. **Please keep this Patient Information and Consent Form** that includes information about your exposure to radiation in this study for at least five years. You will be required to provide this information to researchers of any future research studies involving exposure to radiation

- Questionnaires: no known risks
- Transcranial magnetic stimulation (TMS): in a minority of cases, mild headaches have been reported following exposure to TMS, which can be resolved with widely available over-the-counter analgesics. We will screen all participants with the latest TMS safety guidelines to exclude any individuals that may be at higher risk of adverse effects to TMS.

There may be additional unforeseen or unknown risks.

Expected benefits to the wider community

The findings of this study will help the wider community to treat lower back pain. Back pain is a major public health concern that presents the greatest costs to Australian society, above that of cardiovascular disease, diabetes, cancer and other diseases, in terms of disability and lost productivity. The current study will deliver information that will improve the management of spinal pain conditions.

How privacy and confidentiality will be protected

Any information obtained in connection with this project and that can identify you will remain confidential. It will only be disclosed with your permission, subject to legal requirements.

Forms with identifying information will be stored separately from other study information. For the purpose of treatment and testing appointments, we will use your name in our internal booking systems. Any identifying information stored in electronic form will be stored on a password protected computer.

A unique code will be used on all forms and data collected from you, and not with your name or any other identifying information. These (de-identified) data will be stored on a password protected server.

Only the investigators will have access to the data. Sharing of data with investigators outside of Deakin University will occur only in a coded, anonymised way and no identifying or personal information will be shared. Data collected at Advance Healthcare from participants randomised to the Advance Healthcare group will be shared with the remainder of the research team in a coded manner only.

Your GP will be informed of any health-relevant events that occur or findings (such as from MRI) that are obtained due to your participation in the study.

Otherwise, none of the information provided will be made public in any form that would reveal your identity to an outside party, thus all participants will remain anonymous.

Questionnaires that are filled out online will be completed using only your unique study code. This will ensure we can collate your data internally at Deakin, but your personal identifying information will not be used for these online questionnaires.

Information on paper copy, computer or CD will be stored for 10 years from the date of publication. After a period of 10 years from the date of any publication of the results from the study, paper copies of your individual responses will be disposed of in the interests of limiting physical space taken up by the records. Electronic copies of all data will be retained indefinitely. In accordance with the Freedom of Information Act 1982 (Vic), you have the right to access and to request correction of information held about you by Deakin University.

Dissemination of the research results

If you wish, a brief report via email or the post of your results can be sent to you at the completion of the study. If you express interest in the study results, we can send you copies of published articles via email or post. You can indicate this on the consent form.

The research team will disseminate the findings from this project to relevant agencies and health care professionals that could potentially benefit from these findings. The study results will also be presented at national and international conferences, and will appear in our annual reports and newsletters. The work from this project will also be submitted for publication to international peer-reviewed scientific journals. Should research students become involved in the project, we expect results of the study to appear in their theses. This process will allow the results to be accessed by a large number of health professionals worldwide. Once published, we will also send relevant papers to the key stakeholders in this field.

How the research will be monitored and conducted?

There are a series of scientists involved in this project. The primary co-ordination and implementation of the project will occur at Deakin University. The participant recruitment, telephone screening and physiotherapy treatment group will occur at Advance Healthcare (Boronia). Here we describe the role of each person in the project:

- A/Prof. Daniel Belavy: is responsible for overall study co-ordination and the MRI investigations
- Dr. Jon Ford: is responsible for participant recruitment and implementation of the physiotherapy group
- Dr. Clint Miller: is responsible for the treatment group at Deakin University, study co-ordination, physical function and performance testing, endurance capacity testing, questionnaires, body size and mass
- Dr. Andrew Hahne: is responsible for participant recruitment and implementation of the physiotherapy group

- Dr. Luana Main: is responsible for questionnaire outcomes (motivation, mood, adherence, physical activity) and tools for improving adherence to treatment programmes.
- Dr. Timo Rantalainen: is responsible for DXA examinations and assistance with MRI examinations
- Dr. Wei-Peng Teo: is responsible for TMS examinations
- Dr. Megan Teychenne: is responsible for questionnaire examinations (mood and physical activity)
- Prof. Guy Trudel: is an advisor for the assessment of fat changes in the spine
- Prof. Guoyan Zheng: is responsible for the development of software that analyses the MRI data.
- Prof. Gary Thickbroom: will provide advice on TMS examinations and support in analysis of the TMS data

There will be additional staff and students at the treatment facilities at Advance Healthcare (Boronia) and the Clinical Exercise Learning Centre (CELC) treatment facility who will participate and assist in your treatment.

It is possible that a research student or students may become involved in this project at a later date.

Any payments to participants?

We do not provide payment for your participation in this project, but if you drive to the testing and treatment sessions we can reimburse the parking costs at Deakin University Burwood campus or Swinburne University. Free parking available at Advance Healthcare Boronia for those randomised to that treatment group.

Sources of funding for the research

This research is funded by Deakin University.

Financial or other relevant declarations

The researchers involved in the study do not have any interests, financial or otherwise, that conflict in the conduct of this study.

Participation is voluntary

Participation in this research project is voluntary. If you do not wish to take part, you are not obliged to. If you decide to take part and later change your mind, you are free to withdraw from the project at any stage.

Your decision whether to take part or not to take part, or to take part and then withdraw, will not affect your relationship with Deakin University or Advance Healthcare. You will also have the option to withdraw your data from the research project if you wish to do so.

Before you make your decision, a member of the research team will be available to answer any questions you have about the research project. You can ask for any information you want. Sign the Consent Form only after you have had a chance to ask your questions and have received satisfactory answers.

If you decide to withdraw from this project, please notify a member of the research team or complete and return the Revocation of Consent Form attached. This notice will allow the research team to inform you if there are any health risks or special requirements linked to withdrawing.

Contact details

If you require further information or wish to withdraw your participation from this project, you can contact the principal researcher:

A/Prof Dr. Daniel L. Belavy
School of Exercise and Nutrition Sciences
Deakin University
Melbourne Burwood Campus
221 Burwood Highway
Burwood, VIC 3125

Complaints

If you have any complaints about any aspect of the project, the way it is being conducted or any questions about your rights as a research participant, then you may contact:

The Manager, Ethics and Biosafety, Deakin University, 221 Burwood Highway, Burwood Victoria 3125, Telephone: 9251 7129, research-ethics@deakin.edu.au

Please quote project number [2015-137].

PLAIN LANGUAGE STATEMENT AND CONSENT FORM

TO: Participants

**Consent Form**

Date:

Full Project Title: Optimising conservative management of chronic low back pain

Reference Number: 2015-137

I have read, or have had read to me, and I understand the attached Plain Language Statement.

I understand that I will require written clearance from my GP before beginning with this study.

I freely agree to participate in this project according to the conditions in the Plain Language Statement and can commit to the 6 months course of this project including 2 days of testing every 3 months and the 6 month treatment programme as outlined above.

Please indicate below whether you agree to have your contact details stored to be invited to participate in future research.

☐ Yes, I agree to be invited to participate in future research.

Please tick the box below if you would like to receive a copy of the study results, via email, at the end of the study.

☐ Yes ☐ No

I have been given a copy of the Plain Language Statement and Consent Form to keep.

The researcher has agreed not to reveal my identity and personal details, including where information about this project is published, or presented in any public form.

Participant's Name (printed) ……………………………………………………………………

Signature …………………………………………………… Date ……………………….

Please mail or fax this form to:

A/Prof Daniel Belavy
Deakin University
Centre for Physical Activity and Nutrition Research (C-PAN)
221 Burwood Highway
Burwood, Victoria 3125

Fax: +61 3 9244 6017

PLAIN LANGUAGE STATEMENT AND CONSENT FORM

TO: Participants

**Withdrawal of Consent Form**

(To be used for participants who wish to withdraw from the project)

Date:

Full Project Title: Optimising conservative management of chronic low back pain

Reference Number: 2015-137

I hereby wish to WITHDRAW my consent to participate in the above research project and understand that such withdrawal WILL NOT jeopardise my relationship with Deakin University or Advance Healthcare.

Participant's Name (printed) …………………………………………………….

Signature ……………………………………………………………… Date ……………………

Please mail or fax this form to:

A/Prof Daniel Belavy
Deakin University
Centre for Physical Activity and Nutrition Research (C-PAN)
221 Burwood Highway
Burwood, Victoria 3125

Fax: +61 3 9244 6017
